# Microbial Profiles of Patients With Antipsychotic-Related Constipation Treated With Electroacupuncture

**DOI:** 10.3389/fmed.2021.737713

**Published:** 2021-10-14

**Authors:** Yuanjia Zheng, Xiumin Jiang, Yacen Gao, Lexin Yuan, Xiaotong Wang, Shengwei Wu, Yucen Xia, Lin Yao, Jinglan Yan, Lanying Liu, Yingdong Wei, Zhiqiang Song, Lin Yu, Yongjun Chen

**Affiliations:** ^1^South China Research Center for Acupuncture and Moxibustion, Medical College of Acu-Moxi and Rehabilitation, Guangzhou University of Chinese Medicine, Guangzhou, China; ^2^Rehabilitation Center, The First Affiliated Hospital of Guangzhou University of Chinese Medicine, Guangzhou, China; ^3^Department of Traditional Chinese Medicine, The Affiliated Brain Hospital of Guangzhou Medical University (Guangzhou Huiai Hospital), Guangzhou, China; ^4^Research Institute of Acupuncture and Moxibustion, Shandong University of Traditional Chinese Medicine, Jinan, China; ^5^Department of Psychosomatics, Tongde Hospital of Zhejiang Province, Hangzhou, China; ^6^Medical Administration Division, Shenyang Anning Hospital, Shenyang, China; ^7^The Third People's Hospital of Qinghai Province, Xining, China; ^8^Guangdong-Hong Kong-Macao Greater Bay Area Center for Brain Science and Brain-Inspired Intelligence, Guangzhou, China

**Keywords:** antipsychotic-related constipation, electroacupuncture, spontaneous bowel movements, 16S rRNA gene sequencing, gut microbiota

## Abstract

Antipsychotic-related constipation (APRC) is one of the most common side effects of taking antipsychotic medication. APRC can seriously impact patient quality of life and is potentially fatal, though the efficacy of current APRC treatments is low for most patients. In this study, we conducted a controlled, pilot randomized, sham-electroacupuncture (SEA) study to assess the efficacy of electroacupuncture (EA) in patients with APRC. We used 16S rRNA gene sequencing to assess the microbial profiles of these patients and analyze how EA treatments affected their bacteria.

**Methods:** We treated 133 APRC patients with randomly assigned EA treatments or SEA treatments for 4 consecutive weeks, fully evaluating the patients 8 weeks after treatment. The participants, outcome assessors, and statistics were all blind to the EA and SEA treatments. Outcomes assessed included changes in spontaneous bowel movements (SBMs) and the frequency of rescue measures. We detected assessed the microbial diversity of stool specimens both before and after EA treatment using 16S rRNA gene sequencing.

**Results:** Both EA and SEA treatments reduced the need for constipation rescue measures and did not have serious side effects. EA treatments were better than SEA treatments at increasing SBMs and reducing rescue measures. The diversity of gut microbiota changed after EA treatment. LEfSe analysis indicated changes in the genus (belonging to phylum *Proteobacteria*) of gut microbiota in patients following EA treatment.

**Conclusions:** This study found that EA treatment is effective and safe for patients with APRC, and could be associated with changes in their microbial profiles. Further study, with larger sample sizes, is needed to explore the efficacy of EA intervention as a clinical treatment for APRC.

**Trial Registration:** ChiCTR, ChiCTR-ONC-17010842, http://www.chictr.org.cn/showproj.aspx?proj=18420.

## Introduction

Antipsychotic medications are pharmaceuticals that are widely used to treat various psychiatric disorders. Constipation is a common and potentially fatal side effect of these medications and can seriously affect both patient health and quality of life (1–6). The incidence rate of constipation in patients treated with clozapine is 30–60% (1, 3, 4, 7, 8) and in patients treated with olanzapine is 9–11% (9–11). Antipsychotic-related constipation (APRC) can result in serious complications including intestinal obstruction, colon obstruction, intestinal ischemia and perforation, hepatic venous outflow block, and fatal intra-abdominal sepsis (1, 3, 4, 7–13). Pharmacotherapy has been used to treat APRC, however, its efficacy is limited (6, 14, 15), and there insufficient data about how safe these treatments are (5, 15). For example, laxatives are the most commonly used treatment for constipation, but up to 50% of patients with constipation dislike being dependent on laxatives due to their adverse effects and temporary therapeutic efficacy (16–19). Treatments such as prokinetic medicine have serious drawbacks, including a delayed therapeutic response, the potential for cardiac diseases, and unclear long-term effects (20–24). Given the current state of APRC treatments, safe and effective alternatives are needed.

Acupuncture is one non-pharmacologic alternative. Recent studies have demonstrated that electroacupuncture (EA) is significantly more effective at treating symptoms of constipation than sham EA treatments (25–27). However, the efficacy and safety of treating APRC with EA are still unknown. Previous studies observed disturbance in the intestinal flora of patients with constipation (28–31), while a healthy intestinal microbial environment can facilitate intestinal peristalsis. Patients with constipation may experience changes in their gut microbiota, which are characterized by increases in potentially pathogenic microorganisms and fungi and decreases in obligate bacteria (32, 33). These alterations in the gut microbiota could change the concentration of physiologically active substances that are caused by changes in the metabolic milieu of the colon, which could influence the secretory and motor functions of the bowel (34, 35). For example, studies of animal and human models have demonstrated that methane, which is mainly produced by *Archaea*, can delay intestinal transit (36, 37). In addition, previous studies have demonstrated that intestinal motility could be promoted by altered microbial-derived metabolites such as short-chain fatty and tryptamine acids (38–40). However, few studies have assessed the relationship between microflora and the effects of treating APRC with EA, and it is unclear whether EA treats constipation via microflora.

We designed a randomized, SEA-controlled clinical pilot trial and employed 16S rRNA sequencing to identify multiple microbial profiles. The aims of the study are to evaluate the efficacy and safety of treating APRC patients with EA and to better understand whether EA can treat APRC by regulating intestinal flora.

## Materials and Methods

### Subjects

**Figure 1:**
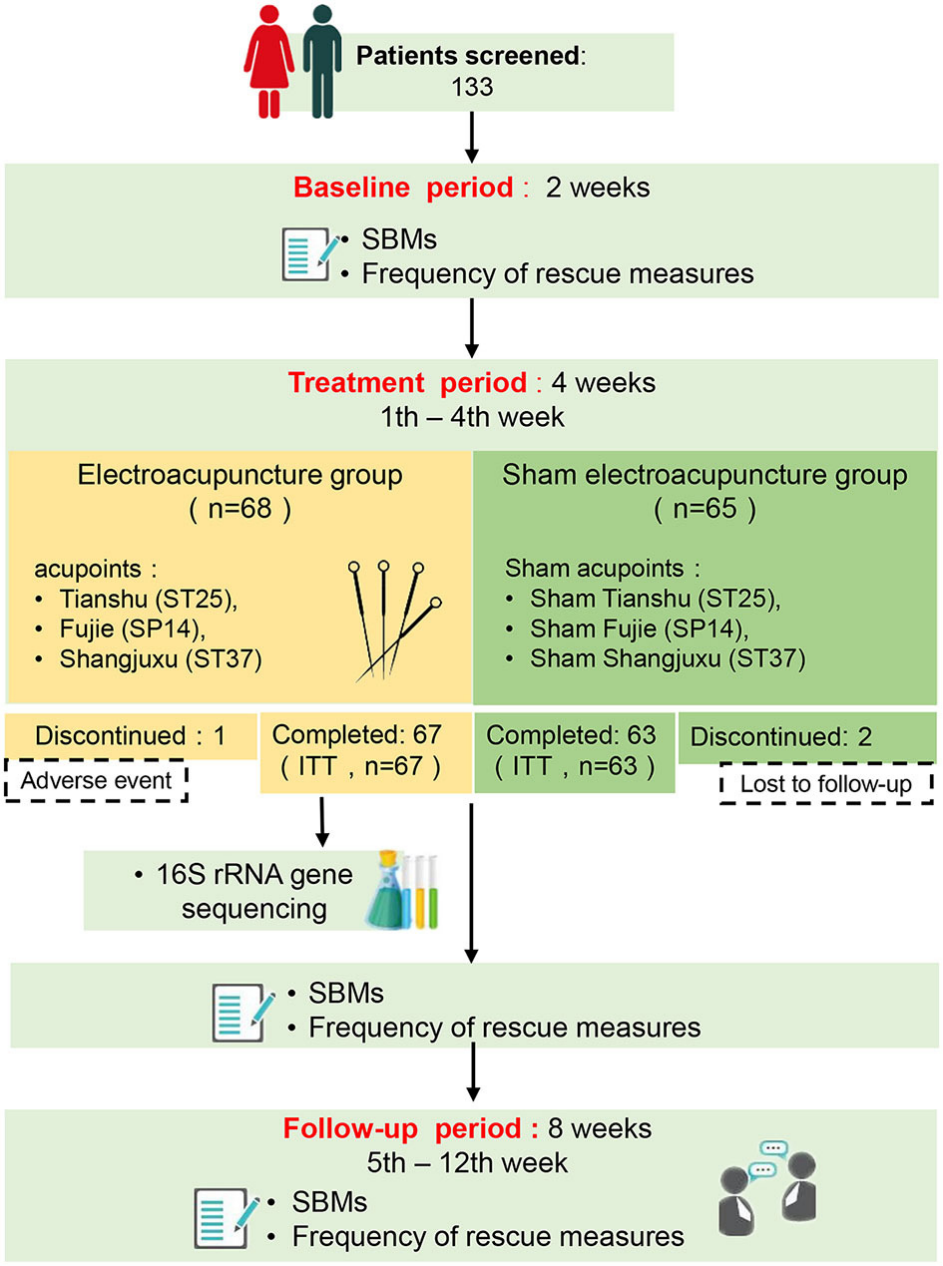
Flow diagram of the study. SBM, spontaneous bowel movements; ITT, intent to treat.

One hundred and thirty-three (133) APRC patients were recruited from March 2017 to October 2020 from four hospitals (Guangzhou Huiai Hospital, Tongde Hospital of Zhejiang Province, The Third People's Hospital of Qinghai Province, and Anning Hospital of Shenyang) in China. All subjects were randomly assigned to either EA treatment or SEA treatment, and 130 completed the study (see Figure 1, flow diagram).

The ethics committees of the hospitals approved the study, and the requisite protocols were registered in the Chinese Clinical Trial Register (ChiCTR, ChiCTR-ONC-17010842, registered 11 March 2017). We adopted the inclusion criteria used in prior APRC studies (3, 16), which includes the following: (1) Subjects ranged in age between 18 and 65 years old and read and understood all trial-related procedures, (2) No constipation reported prior to the use of antipsychotic drugs, (3) Subjects were diagnosed with mental disorders using the Diagnostic and Statistical Manual of Mental Disorders (Fifth Edition), had consistently been taking antipsychotics for at least 3 months, and developed constipation as a side effect of taking antipsychotic medication, (4) Subjects received no acupuncture treatment and did not participate in any other constipation trials over the previous 3 months, (5) Subjects included in this trial meet the Roman III diagnostic criteria for functional constipation, the specific diagnostic criteria was presented in Supplementary Material 1.

Patients with the following criteria were excluded from the study: (1) Constipation caused by other drugs or non-antipsychotic pharmaceuticals, (2) Blood coagulation disorders, (3) The presence of tumors, (4) Serious organ-related illnesses, including severe cardiovascular, hepatic, renal diseases, or cardiac pacemaker implantation, (5) Breastfeeding or pregnancy, (6) Any other condition that might interfere with the operation and results. The study met the requirements of the Declaration of Helsinki, the International Conference on Harmonization Good Clinical Practice E6 guidelines, and the Standards for Reporting Interventions in Clinical Trials of Acupuncture guidelines.

### Procedures

Participants provided informed consent and were randomly placed into either the EA or Sham EA (SEA) treatment group. Their baseline characteristics were evaluated 2 weeks before intervention and they received 12 sessions of EA/SEA treatment over the course of 4 weeks. All patients were monitored for 8 weeks following treatment (see Figure 1, flow diagram). An equal number of patients were assigned to either the EA group or the SEA group according to a randomized block design generated by an independent statistician using SAS 9.4 with block length 4 and study site as a stratified variable. Randomization sequences were maintained and concealed by a statistician, who was blind to the group assignment and other procedures during the course of the study. For each patient, the acupuncturist was assigned a randomized number from the statistician via mobile text, which assigned the patient to one of the two groups. Sufficient random numbers were established to take account of subjects' loss of follow-up and withdrawal. Except for the acupuncturist, all parties, including patients, were unaware of their group assignment. The acupuncturists, outcome assessors, and statisticians were independent of each other and did not communicate over the course of the study to ensure the study was blind. A blinding test was conducted during treatments performed in the second and fourth week by asking the participants whether they were receiving EA treatment, SA treatment, or an unknown treatment.

### Intervention

The EA treatment was developed from traditional Chinese acupuncture based on the advice of acupuncture experts and published studies concluding that EA can effectively treat constipation (25–27). The duration of EA/SEA treatment sessions was as follows: each treatment was 30 min long, three sessions were conducted each week, and 12 sessions were conducted over 4 weeks. This trial used disposable 0.30 × 50–mm or 0.35 × 75–mm needles (Huatuo, Suzhou, China) and EA/SEA devices (HANS-200, Nanjing, China). The treatment was free for participants. *EA group:* After sterilizing the skin, the acupuncturist inserted the needles into the acupoints [bilateral Tianshu (ST25), Fujie (SP14), and Shangjuxu (ST37)] (Supplementary Figure 1) slowly at a 90° angle. The puncture depth of the abdomen acupoints was ~15–25 mm and the puncture depth of the limb acupoints ~15–20 mm. The needles at the abdomen acupoints were subsequently attached to an EA device (KWD-8081, YINGDI, Changzhou, China), which emitted 10/50 Hz dilatational waves for 30 min at a current intensity of 0.1–1 mA. The intensity of the current was adjusted to meet the patients' comfort level. *SEA group*: The acupoints of the SEA group were bilateral sham ST25, sham SP14, and sham ST37, which were located 2 cm to the side to the acupoints of the EA treatment. The needles of the SEA group were superficially inserted ~2–3 mm into the skin. The SEA device had the same light signals as the EA group, but with no electricity. Depending on the clinical situation, a laxative rescue measure was used as a rescue treatment for participants who did not defecate for 3 or more days. No other drugs taken during treatment except antipsychotics drugs (see Table 1) and basic laxative rescue measures (Kayseri and Folium sennae).

**Table 1:** Summary of patient demographics and baseline characteristics.

	**Electroacupuncture group (*n* = 67)**	**Sham electroacupuncture group (*n* = 63)**	***P*-value**
Age (years), mean (range)	36.36 ± 14.31	39.22 ± 13.48	*P* > 0.05
**Gender**			
Male	18 (27%)	21 (33%)	*P* > 0.05
Female	49 (73%)	42 (67%)	*P* > 0.05
**Basic line**			
Average of course (year)	10.53 ± 10.85	12.21 ± 10.51	*P* > 0.05
Average of SBMs per week (*n*)	2.96 ± 1.73	2.56 ± 2.13	*P* > 0.05
Frequency of rescue measures per week (*n*)	1.38 ± 0.66	1.43 ± 0.75	*P* > 0.05
**Antipsychotic drugs use**, ***n*** **(%)**			
Clozapine	16 (24%)	15 (24%)	*P* > 0.05
Olanzapine	25 (37%)	23 (37%)	*P* > 0.05
Risperidone	21 (31%)	21 (32%)	*P* > 0.05
Quetiapine	13 (19%)	14 (23%)	*P* > 0.05
Aripiprazole	4 (6%)	4 (6%)	*P* > 0.05
Others	10 (15%)	4 (6%)	*P* > 0.05

*SBM, spontaneous bowel movements*.

### Outcome

Changes in spontaneous bowel movements (SBMs) were used to assess primary outcomes. Changes in SBMs were considered by assessing the mean weekly SBMs over the course of the treatment period (4 weeks) or follow-up period (8 weeks), minus the mean weekly SBMs of baseline (2 weeks). The average SBMs at baseline (2 weeks) and during the treatment period (4 weeks) were considered pre-and post-treatment values, respectively. All participant defecation conditions were recorded by research assistants. SBMs were considered to be complete or incomplete defecation occurring without the help of any medicine or other method over the previous 24 h. Secondary outcomes were considered by assessing the frequency of rescue measures used, which, for participants who had no defecation for 3 or more days, was typically an enema.

### Sample Size

This pilot trial was a preliminary exploration of the potential efficacy of treating APRC patients with EA. The basis for our experimental design included assessing the increase of SBMs in the EA and SEA groups during the follow-up period of a previous study assessing how EA affects severe constipation (25). We calculated that 27 patients per group were needed in this pilot study to achieve an alpha risk of 0.05. After accounting for a 15% dropout rate, sample size was set at 32 participants for each group, though we recruited 133 participants, 130 of whom completed the study.

### 16S rRNA Analysis of Fecal Samples

Feces were collected at hospital sites, divided into 200 mg parts, and stored at −80°C until extraction. Total genome DNA was extracted from stool samples via the Cetyltrimethylammonium Bromide method. Agarose gel electrophoresis was performed to detect DNA purity and concentration. Combined with adapter sequences and barcode sequences, the V3–V4 region of the bacterial 16S rRNA gene was amplified with the common primer pair (Forward primer, 5′- CCTACGGGRBGCASCAG-3′; reverse primer, 5′- GGACTACNNGGGTATCTAAT-3′). The PCR products were purified using a MinElute Gel Extraction Kit (Qiagen, Germany). Sequencing libraries were generated using a TruSeq® DNA PCR-Free Library Preparation Kit (Illumina, USA), and libraries were sequenced using the Illumina NovaSeq6000 system (Novogene, China) following quantification by a Qubit 2.0 Fluorometer (Thermo Scientific, USA). To obtain clean tags, raw fastq files were merged using FLASH V1.2.7 (41). VSEARCH (42) was used to assess effective tags and remove the chimera sequence. Using Uparse 7.0 (43), operational taxonomic units (OTUs) were clustered with sequence similarities ≥97%. Taxonomic information was annotated using the MUSCLE (Version 3.8.31) (44) according to the Silva database [http://www.arb-silva.de/; (45)]. Information regarding OTU abundance was normalized using a standard sequence number corresponding to the sample with the least sequences. Subsequent analysis of alpha diversity was performed based on the resulting normalized data. The estimate of richness index was calculated according to OTU abundance. In the alpha diversity index, the Chao, Shannon, and phylogenetic diversity indexes were calculated via Qiime (Version 1.9.1) (46). The random forest package in R was used to build the prediction model and identify potential therapeutic targets. Model training was generated from 80% of the sample set. The core genera in the pre- or post-treatment groups were filtered as prediction input variables. The important genera contributing to the prediction were performed by a nested 10-fold cross-validation procedure. The efficiency of possible cutoff values was predicted by conducting a receiver operating characteristic (ROC) analysis and assessing the area under curve (AUC) index. The raw data were deposited onto NCBI's SRA database and the accession number is PRJNA735596 (https://www.ncbi.nlm.nih.gov/sra/PRJNA735596).

### Data Analysis

All randomized patients who received at least 4 weeks of treatment were included in the intent-to-treat (ITT) population. A chi-square test was used to assess categorical variables with SPSS 22 (IBM, USA). Student's *t*-test and two-way analysis of variance (ANOVA) were used to test each variable for differences between two groups using Prism 7 (GraphPad Software, USA). A two-tailed Mann-Whitney test was used to evaluate statistical significance in non-parametric tests. Welch's correction was performed if the analysis of variance was found to have a significant effect. Wilcoxon signed-rank test was used to analyze the difference between groups in the Alpha diversity index. A Kruskal-Wallis test and a permutation test were used to evaluate the statistical significance of relative abundance. To identify a differential representative of taxa and functional modules between before and after EA treatment, a linear discriminant analysis effect size (LEfSe) analysis was used to identify differentially abundant taxa modules [those with linear discriminant analysis (LDA) score >3.0]. A Spearman's rank correlation analysis was performed on the bacterial taxa and the SBMs score to assess the relationship between disease severity and gut microbiota. The correlation analysis was only performed in those genera generated from the random forest model and those class, phylum, family genera were assessed via Lefse. All values are displayed as the mean ± standard error. *p* ≤ 0.05 were considered statistically significant.

## Result

### Dropout

This study originally recruited 133 patients, all of whom were randomly assigned to two groups. Of them, 130 completed the study (see Figure 1, flow diagram). One patient in the EA group withdrew from the study due to adverse reactions during EA treatment, and two patients in the SEA group were removed from the study because we were unable to follow up with them. The missing data (*n* = 3) was imputed using a multiple imputation approach.

### Baseline Comparison

The general characteristics of APRC patients are summarized in Table 1. There were no differences between the two groups in terms of age, gender, antipsychotics, average course of treatment, mean SBMs per week, or frequency of enema use.

### Primary Outcome

**Figure 2:**
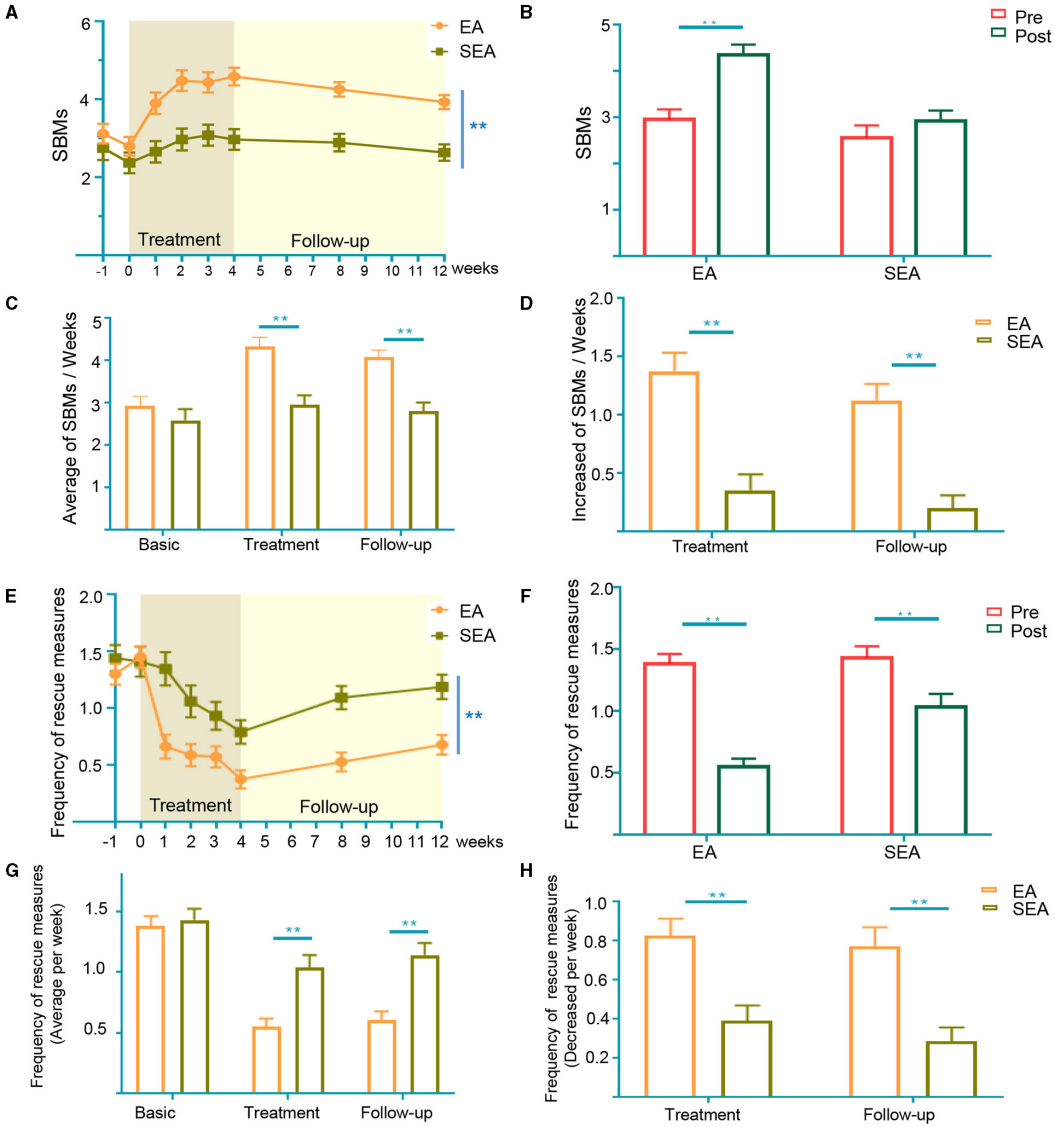
Changein SBMs and frequency of enema uses per week. **(A)** SBMs per week in the entire trial. Two-way analysis of variance (ANOVA); **(B)** SBMs in pre- and post-treatment period. The data of pre-treatment generated from the average of the basic period (−1 and 0 week in **A**). The data of post-treatment generated from the average of the treatment period (1–4week in **A**); **(C)** mean weekly SBMs at baseline, treatment period and follow-up period; **(D)** increase in SBMs of treatment period and follow-up period from baseline; **(E)** frequency of enema uses per week in the entire trial. Two-way analysis of variance (ANOVA); **(F)** frequency of rescue measures in pre- and post-treatment period. The data of pretreatment generated from the average of the basic period (−1 and 0 week in **A**). The data of post-treatment generated from the average of the treatment period (1–4week in **A**); **(G)** mean weekly frequency of enema uses at baseline, treatment period, and follow-up period; **(H)** decrease in frequency of laxative rescue measures uses during treatment and follow-up periods from baseline. SBM, spontaneous bowel movements. Error bar is presented as mean ± standard derivation error, Baseline: 2 weeks (Week −1 to Week 0). Treatment: 4 weeks (Week 1 to Week 4). Follow-up: 8 weeks (Week 5 to Week 12). *p < 0.05, **p < 0.01, Mann Whitney test.

During the trials, we analyzed the number of SBMs per week to assess the therapeutic efficacy of the EA treatment. As shown in Figure 2A, the weekly SBMs of both groups increased during the trial period. Compared to SEA treatment, EA treatment was more effective at treating APRC both during the treatment period and during the follow-up period (*p* < 0.01). EA treatment could significantly improve SBMs after treatment (*p* < 0.01), while there was no significant difference before or after SEA treatment (Figure 2B). The weekly SBMs during the treatment period and the follow-up period of the EA group were 4.34 ± 1.81 and 4.08 ± 1.43, respectively, while the weekly SBMs during the treatment period and the follow-up period of the SEA group were 2.91 ± 1.79 and 2.76 ± 1.66, respectively. The number of mean weekly SBMs increased during the EA treatment period and subsequent follow-up periods compared to the SEA group (*p* < 0.01; Figure 2C). The number of SBMs per week in the EA group during the treatment period and follow-up period were 1.39 ± 1.26 and 1.13 ± 1.11, respectively, while the number of SBMs per week in the SEA group was 0.36 ± 1.09 and 0.20 ± 0.84, respectively. The average SBMs of the EA group had a higher weekly increase compared with the SEA group during both the treatment period and the follow-up period (Figure 2D).

### Secondary Outcome

The frequency of weekly laxative rescue measures use decreased in both groups during the trial period. Over 12 weeks, the weekly change from the baseline rescue measures was similar to SBMs, though patients receiving EA treatment saw decreases in the frequency of their enema uses (*p* < 0.01; Figure 2E). Both EA and SEA treatments had a significantly lower frequency of rescue measures compared to the baseline (Figure 2F). The weekly frequency of enema usage during the treatment and follow-up period of the EA group was 0.54 ± 0.53 and 0.59 ± 0.58, respectively, while the weekly frequency of enema usage during the treatment and the follow-up period of the SEA group was 1.03 ± 0.83 and 1.14 ± 0.77, respectively. The frequency of rescue measures was significantly lower in the EA group during both the treatment and follow-up period (*p* < 0.01; Figure 2G). The number of enemas per week in the EA group decreased during the treatment period and the follow-up period by 0.83 ± 0.71 and 0.78 ± 0.79, respectively, while the number of enemas per week in the SEA group decreased by 0.40 ± 0.62 and 0.28 ± 0.57, respectively. The frequency of enema use per week decreased more in the EA group than in the SEA group during both the treatment period and the follow-up period (*p* < 0.01; Figure 2H).

### Adverse Events

Only one patient in the EA group experienced a subcutaneous hematoma, which was mild and transient.

### Blinding Assessment

In the EA group, 80.65% of participants correctly guessed that they were receiving EA treatment when they were asked in the second week, and 74.19% guessed correctly in the fourth week. In the SEA group, 73.33% of participants correctly guessed that they were receiving SEA treatment when asked at the second week and 66.67% guessed correctly at the fourth week. No statistical difference was found between the two groups.

### Changes in the Composition of Gut Microbiota Before and After Treatment Based on 16S rRNA Data

**Figure 3:**
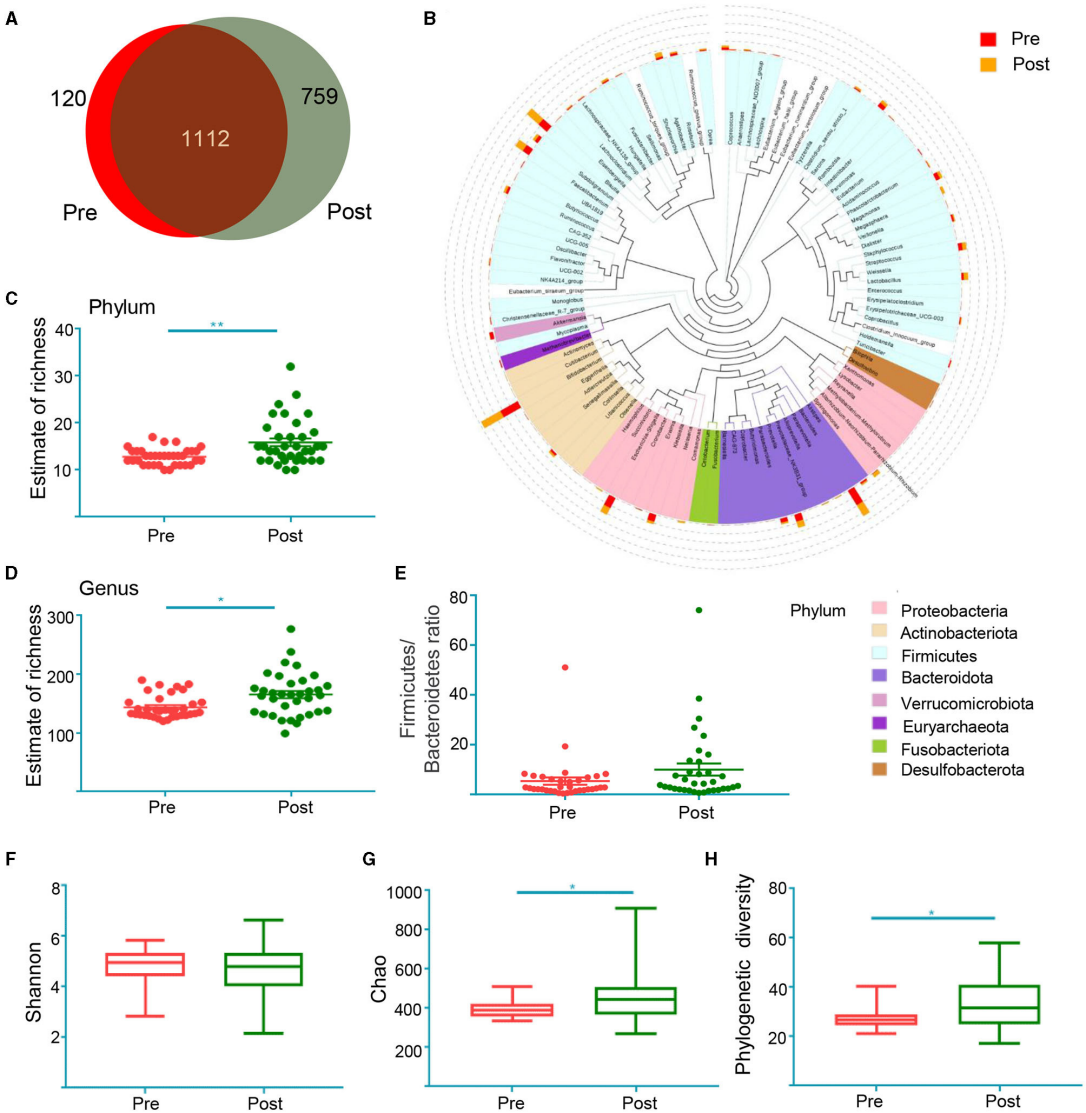
The shift of gut microbiota in the patients before treatment (Pre) and after treatment (Post) according to the 16S rRNA data. **(A)** Venn diagram of the observed OTUs in Pre and Post; **(B)** the representative sequences of Top 100 were obtained by multi-sequence alignment. The colors of branching and fan represent the corresponding phylum, and the stacking bars outside of the fan ring indicate the abundance distribution information of the genus in different samples; the estimate of richness index analysis between two groups at the level of Phylum **(C)** and genus **(D)**. *p < 0.05, **p < 0.01, Mann Whitney test; *Firmicutes–Bacteroidetes* ratio at the phylum level **(E)**. Alpha diversity indices of Shannon index **(F)**, Chao index **(G)** and phylogenetic diversity index **(H)**. *p < 0.05, **p < 0.01, Wilcoxon rank-sum test.

Fecal samples were collected from 35 patients before and after EA treatment to assess their 16S rRNA sequence. Effective tags ranging from 47,599 to 75,487 were obtained from all samples (Supplementary Material 3). Rarefaction curves were calculated according to the OTUs (Operational Taxonomic Units) and demonstrated high rates of sampling coverage (~99%) (Supplementary Figure 2), indicating that their sequencing depth was adequate to investigate their gut microbiota. A Venn diagram displays 120 unique OTUs pre-treatment and 759 unique OTUs post-treatment, with 1,112 OTUs shared by both treatments (Figure 3A). To determine the phylogenetic relationships of species at the genus level, we obtained the representative sequences of the top 100 OTUs via multi-sequence alignment (Figure 3B). As shown in Figures 3C,D, richness at the phylum level and the genus level was significantly higher after treatment than before treatment. Considering that the imbalance of the *Firmicutes–Bacteroidetes* ratio may be closely related to the disease such as inflammatory bowel disease, type 2 diabetes and obesity (47–49), we compared the *Firmicutes–Bacteroidetes* ratio at the phylum level. This allowed us to compare the ratio pre-treatment with the ratio post-treatment (Figure 3E). The Shannon index demonstrated no significant changes post-treatment compared to pre-treatment (Figure 3F), but the Chao index demonstrated that post-treatment changes increased compared to pre-treatment (Figure 3G). To measure the degree of evolutionary divergence between the two groups, we assessed the phylogenetic diversity (PD) index. The PD index was higher after treatment than before treatment, which was similar to Chao (Figure 3H). These results demonstrate that the diversity of gut microbiota after treatment was significantly different.

### Therapeutic Target Prediction of EA for Antipsychotic-Related Constipation Based on Gut Microbiota

**Figure 4:**
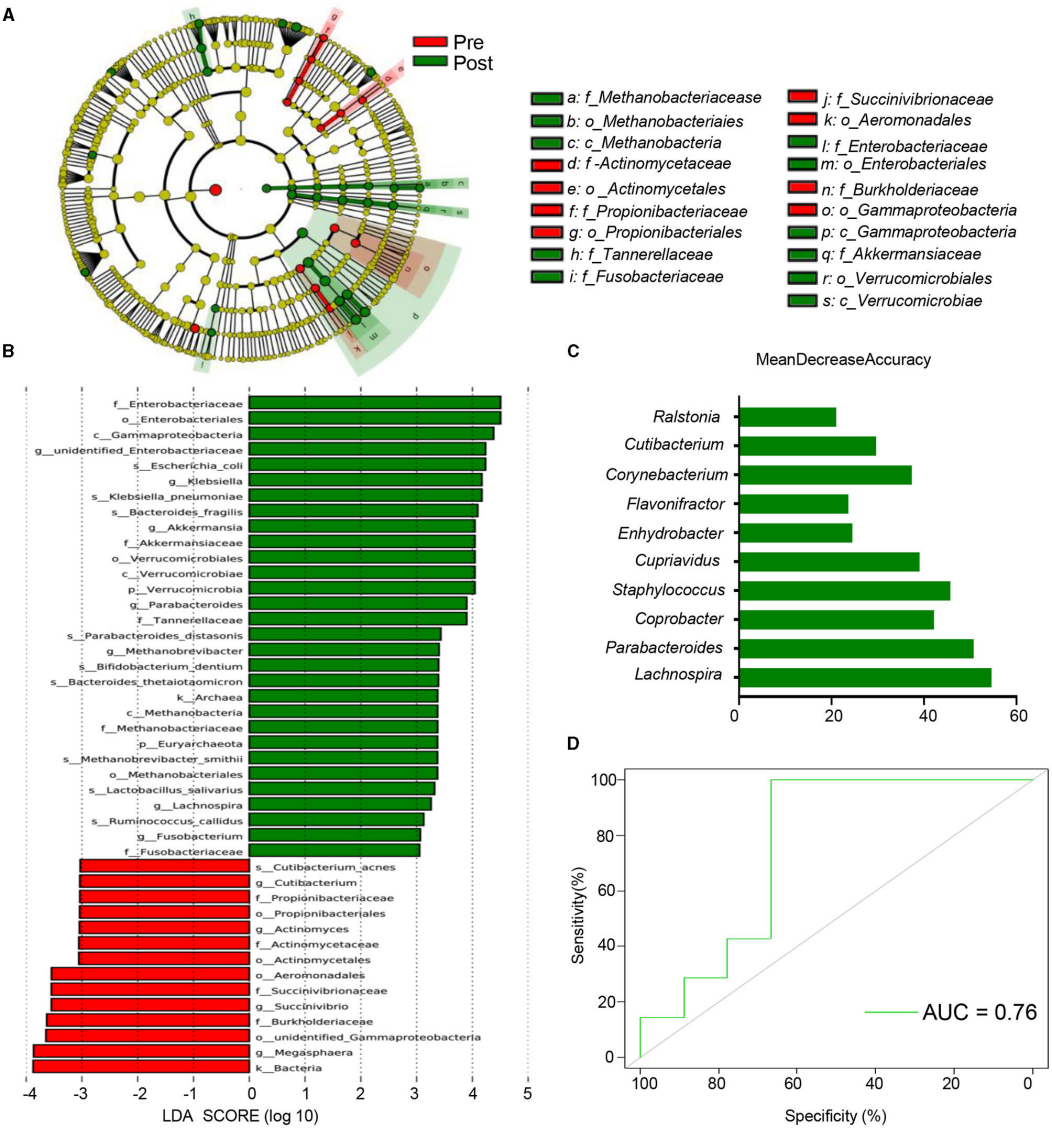
Multiple possible targets for EA of antipsychotic-related constipation. **(A)** Cladograms generated by LEfSe indicating differences in the bacterial taxa between pre and post. Red bars indicate taxa were enrichment in Pre, green bars indicate taxa were enrichment in post. The p_, c_, o_, f_, g_, s_ represent phylum, class, order, family, genus, and species, respectively; **(B)** LDA scores for the bacterial taxa differentially abundant between pre and post (LDA >3). Green bars indicate taxa were enrichment in post, and red bars indicate taxa were enrichment in pre; **(C)** the 10 most discriminant genera in the models classifying pre and post, which identified by machine learning using the random forest analysis. The bar lengths indicate the importance of the variable. The colors represent enrichment in pre (red) or post (green); **(D)** ROC curve displaying the classification for pre and post-employing 16S rRNA data. AUC, area under curve.

A Kruskal-Wallis test was performed to identify the different taxa and functional modules present both before and after EA treatment, which found that a total of 74 genera demonstrated differential relative abundance between the two groups (Supplementary Material 4). Differential microbiota were identified via LEfSe analysis. The phylogenetic branch tree diagram displays the microbial community or species structure and the differences between groups at each level (Figure 4A). An analysis of the distribution diagram (LDA score >3) indicated that microbiotal changes were characterized by higher levels of *Gammaproteobacteria, Enterobacteriales, Enterobacteriaceae*, and *Klebsiella* (which all belong to *Proteobacteria*) in post-treatment participants, while the genera *Megasphaera, Burkholderiaceae, Succinivibrio*, and *Aeromonadales* were more abundant in pre-treatment participants (Figure 4B). These results suggest that EA affects microbiota related to the relief of constipation symptoms.

We developed a random forest model, based on the genus, to determine whether potential biomarkers can be used to predict therapeutic targets. A 10-fold cross-validation method was used to identify representative variations, which we used to analyze the most important changes between the two groups. The optimal model 10 genera that were able to assess changes between the two groups, including members of the *Lachnospira, Parabacteroides, Coprobacter, Staphylococcus*, and *Cupriavidus* genera (Figure 4C). Ten genera could determine whether patients had already been subjected to treatment at an AUC of 0.76 (Figure 4D). These results provide a reference for candidate bacteria instrumental in EA therapy.

**Figure 5:**
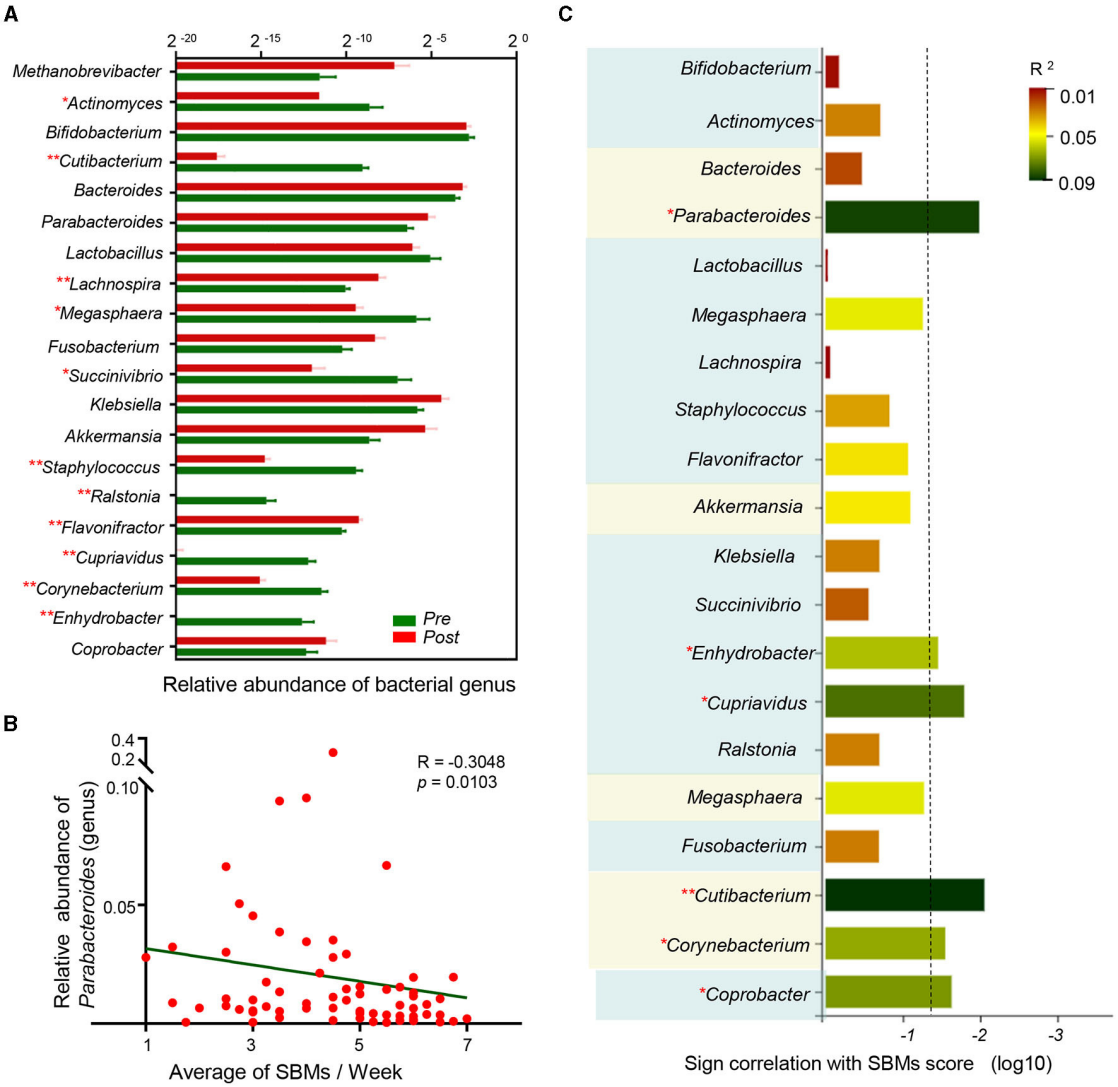
Multiple targets reflect antipsychotic-related constipation severity. **(A)** Column diagram showed relative abundance of discriminant genera by random forest analysis and LEfSe across two groups. *p < 0.05, **p < 0.01, permutation test; **(B)** Spearman correlation between the average of SBMs and relative abundance of *Parabacteroides* (genus); **(C)** sign of the Spearman correlation between bacterial taxa and the average of SBMs. Horizontal axis represents the significance of Spearman correlation and the column surpasses the broken line means *p* < 0.05. Vertical axis represents the taxa include discriminant genera by random forest analysis and the bacterial taxa generated by LEfSe. The taxa belonged to the same phylum have been marked with the same background color. Colors represent the *R*^2^.

Most of the relative genera abundances, which were generated from LefSe and the random forest model, significantly changed following treatment (Figure 5A). We then explored the correlation between the microbiota and the SBMs score, which indicated disease severity. A Spearman's rank correlation analysis demonstrated that six bacterial taxa (*Parabacteroides, Enhydrobacter, Cupriavidus, Cutibacterium, Corynebacterium*, and *Coprobacter*) were correlated with constipation severity (Figures 5B,C). Together, these results indicate that the specific microbial patterns of *Enhydrobacter, Cupriavidus, Cutibacterium, Corynebacterium*, and *Coprobacter* could be potential therapeutic target for EA to treat antipsychotic-related constipation.

### Characteristics of Gut Microbiota Composition in Different Age Groups

**Figure 6:**
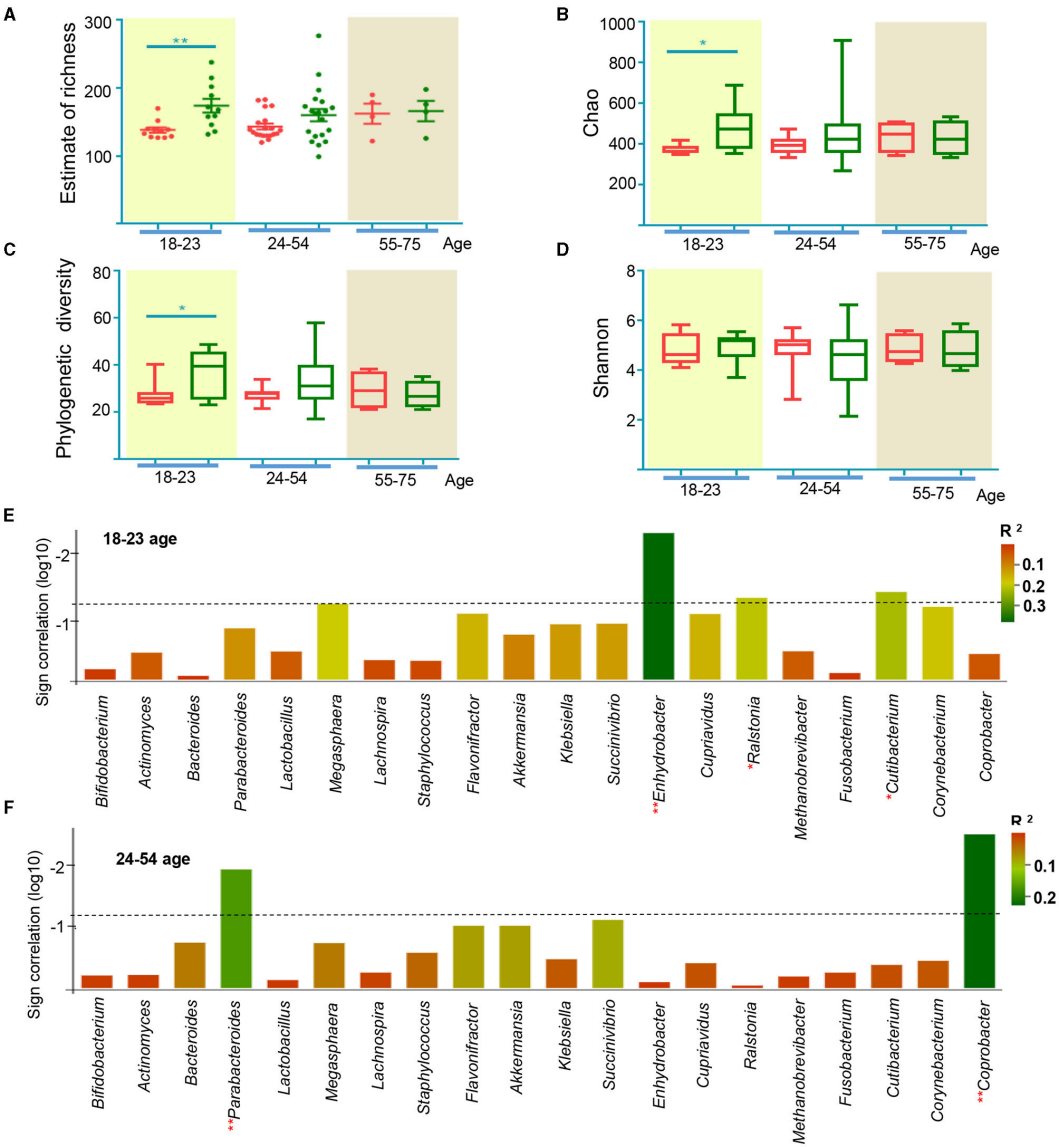
Gut microbiome characteristics according to age from 18 to 23, 24 to 54, and 55 to 75. **(A)** The estimate of richness index analysis between age from 18 to 23, 24 to 54, and 55 to 75 at the level of and genus. *p < 0.05, **p < 0.01, Mann Whitney test; alpha diversity indices of Chao index **(B)**, phylogenetic diversity index **(C)**, and Shannon index **(D)** according to age from 18 to 23, 24 to 54, and 55 to 75. *p < 0.05, **p < 0.01, Wilcoxon rank-sum test; sign of the Spearman correlation between bacterial taxa and the average of SBMs according to age from 18 to 23 **(E)**, 24 to 54 **(F)**. Vertical axis represents the significance of Spearman correlation. The column surpasses the broken line means *p* < 0.05. Horizontal axis represents the taxa include discriminant genera by random forest analysis and the bacterial taxa generated by LEfSe. Colors represent the *R*^2^.

Previous studies have demonstrated that intestinal flora is different at different ages (50), leading us to analyze participant microbial profiles according to their ages (51). The effect of EA treatments on APRC patients was similar for patients in the 18 to 23-and 24 to 54-year-old age groups (Supplementary Figure 3). We found that EA treatments induced significant changes in patients aged 18–23, while EA treatments did not produce any changes in patients aged 24–54 for Estimate of richness (Figure 6A). Analyses of the Chao index, Shannon index, and phylogenetic diversity found similar changes in these groups (Figures 6B–D). Compared to Figure 5C, the correlation between microbiota and constipation severity differed in various groups of APRC patients (Figures 6E,F). *Enhydrobacter, Ralstonia*, and *Cutibacterium* were correlated with constipation severity in the 18 to 23-year-old age group, while no association was found in the 24 to 55-year-old age group. However, *Parabacteroides* and *Coprobacter* were more closely correlated with constipation severity in the 24 to 55-year-old age group, while a weak correlation was found in the 18 to 23-year-old age group. These results demonstrate that the compositional development of gut microbiota in APRC patients aged 18–23 was more sensitive to EA treatment. Altogether, these analyses suggest that the mechanism behind EA treatments could be associated with imbalanced gut microbiota in different age groups.

## Discussion

To our knowledge, this is the first randomized, sham-controlled, clinical pilot trial to evaluate the efficacy and safety of treating APRC patients with EA. This study demonstrated that both EA and SEA treatments reduced the rescue measures needed to relieve constipation (Figure 2F). EA treatments were more effective than SEA treatments at increasing SBMs and reducing rescue measures, particularly during the 8-week follow-up period (Figures 2A,E). Our results suggest that changes in specific microbial patterns associated with APRC symptoms could be treated by EA.

This study had a large sample size (130 participants) and demonstrated that treating constipation with EA was more effective than using SEA, and that these therapeutic effects could be sustained for the 12 weeks of the follow-up period, which were consistent with the previous report (27). Similarly, another randomized control trial found that EA treatment resulted in clinically significant improvements in patients with constipation. These improvements exceeded those observed in SEA treatments, and were maintained for 4 weeks after the treatments ended (52). Furthermore, a previous randomized control trial assessing the effects of EA on constipation demonstrated that EA treatment is equally effective when shallow needling is used at real acupoints, and that the sustained effects of both EA treatment and shallow needling are better than the therapeutic effects of lactulose (53). These results indicate that EA is more effective than SEA at treating constipation, and has a sustained effect. Previous studies have demonstrated that the effects of treating some diseases with acupuncture, including pain and post-prandial distress syndrome, can be sustained for long periods (54, 55). This study determined that EA treatment is safe for APRC patients. Current treatments for APRC, such as laxatives, work quickly but are temporary and can cause diarrhea, gastrointestinal discomfort, and drug dependence (16–18, 23). Treatments such as prokinetic medicine may result in side effects such as cardiac disease, and the long-term therapeutic effects remain unclear (20–22, 24, 56). While EA treatments are not immediately effective, they are stable, safe, and provide sustained benefits, suggesting that EA could be a better choice for APRC patients who are not satisfied with laxatives and prokinetic drugs.

Intestinal microbiota maintains the stability of the intestinal microecological environment, metabolism, and contributes to overall health (57). The intestinal microecological environment becomes imbalanced when the diversity and abundance of the microbiota decrease and the proportion of microbiota is unbalanced (58). Previous studies performed on animals have suggested that EA could relieve the symptoms of certain diseases by adjusting the diversity or restoring the structure of the microbiota (59, 60). However, few clinical trials have assessed how EA or acupuncture affects microbiota, especially for patients with constipation. In this study, we observed notable changes in the microbial profiles of patients following EA treatment (Figure 3). Patients aged 18–23 were more sensitive to acupuncture than patients aged 24–54 (Figure 6), while EA treatment significantly affect both APRC patients aged 18–23 and 24–54 (Supplementary Figure S3). Previous studies have demonstrated that age is an important factor affecting the constitution of intestinal microorganisms (61). As patients grow older, the abundance of gut microbiota decreased and the diversity index significantly differed from younger age groups (50). Moreover, the patient's genetic heritage, health, dietary habits, sex, social background, and living environment all influence the diversity and colonization ability of gut microbiota (62–64). This could explain the different responses to acupuncture treatment observed in different age groups.

We identified post-treatment changes in the abundance of some genera, including *Parabacteroides, Enhydrobacter, Cupriavidus, Cutibacterium, Corynebacterium*, and *Coprobacter*, all of which were associated with disease severity (Figures 5, 6). Previous studies have demonstrated the beneficial effects of *Parabacteroides* on metabolic disorders (65). *Parabacteroides* is a potential probiotic (66) and differences in these levels were observed in children with chronic functional constipation (67). We found a correlation between constipation and *Parabacteroides* abundance for patients aged 24–55, indicating that EA treatment could influence *Parabacteroides*, resulting in anti-constipation effects in APRC patients at aged 24–55. *Enhydrobacter* was also related to disease severity, particularly for patients aged 18–23. While some studies have assessed the role of *Enhydrobacter* in intestinal diseases, such as intestinal metaplasia (68), pancreatic head cancer (69), or *Helicobacter pylori* infections (70), it is still unclear whether *Enhydrobacter* contributes to constipation. *Ralstonia*, which, like *Enhydrobacter*, belongs to the phylum *Proteobacteria*, and was correlated with the SBMs score in patients aged 18–23. *Proteobacteria* levels significantly decreased in patients with constipation (71), and rectal samples from patients with irritable bowel syndrome demonstrated changes in the abundance of *Ralstonia* (72). Therefore, further study is required to assess whether *Enhydrobacter* and *Ralstonia* are affected by EA treatment in patients aged 18–23. Altogether, these results provide a framework for understanding the pathogenic mechanism behind APRC and inform subsequent EA treatments for patients of various ages.

Increasing evidence demonstrates that acupuncture can regulate the imbalance of gut microbiota, which could be related to the following mechanisms. First, the microbiota-gut-brain axis appears to be critically involved in the effects of acupuncture by regulating microbiota. Acupuncture stimulation could help to release adenosine, histamine (73, 74) and change the expressions of TRPV1 and pERK (75, 76) in the muscle layers or skin, which activate neurotransmission to the central nervous system. The signal transmitted and generated by the stimulation of the efferent vagus nerve affected the brain (77). The peripheral anti-inflammatory response can be mediated by the activated efferent vagus nerve, resulting in regulating the changes of microbial composition (78, 79). Secondly, the alteration of microbiota may be associated with acupuncture intervention by immune regulation. Th17 and Treg cells are two types of CD4+ T cells. It has also been demonstrated that acupuncture can restore the Treg/Th17 axis (80, 81). The functioning of T cells is affected by gut microbiota (82–84), though it is unclear whether acupuncture regulates gut microbiota via T cells. Therefore, it is necessary to obtain a more detailed mechanism of the microbial profiles induced by acupuncture in patients of APRC.

While this study is the first to provide insights into the relationship between APRC and EA treatments, there are several noteworthy limitations. First, environmental factors and diet were not evaluated before hospitalization. Diet plays an important role in the community structure and function of gut microbiota (85). To minimize this bias, we recruited participants from inpatients, which have similar dietary and hospitalization environments. Second, our results did not identify the microbial profiles of patients from the SHAM group. To minimize the influence of individual heterogeneity on the research results, we compared the pre-and post-treatment samples from the same patient to eliminate the heterogeneity between individuals in the current study. As such, the microbial profiles of the SHAM group are worthy of further investigation. Third, the small sample size in the current work could lead to a higher variability limiting the validity of the results. Therefore, future studies with large sample sizes are needed to validate the conclusions of this study.

## Conclusion

This study demonstrated that EA treatment is safe and effective, that its effects are sustained over the course of the follow-up period, and that it is superior to SEA treatment in treating APRC patients. This study also provided insights into the relationship between the fecal microbiome and EA treatment in APRC patients, suggesting that APRC can be treated using EA to target specific microbiota.

## Data Availability Statement

The original contributions presented in the study are publicly available. This data can be found here: https://www.ncbi.nlm.nih.gov/sra/PRJNA735596.

## Ethics Statement

The studies involving human participants were reviewed and approved by the Ethics Committees of Guangzhou Huiai Hospital, Tongde Hospital of Zhejiang Province, Third People's Hospital of Qinghai Province, and Anning Hospital of Shenyang. The patients/participants provided their written informed consent to participate in this study.

## Author Contributions

YC conceived and designed project. XJ, YG, and LYua collected samples. YZ, XJ, and XW did experiments and analysis. YZ and XJ prepared figures. YC, YZ, XJ, and YG prepared and finished the manuscript. SW, LL, YW, ZS, LYu, YX, LYao, and JY helped revise the manuscript. All authors read and approved the final manuscript, performed data analyses, and interpretations.

## Funding

This work was supported, in part, by National Key R&D Program of China (2019YFC1712105), National Natural Science Fund of China (81973948), Guangdong Province Universities and Colleges Pearl River Scholar Funded Scheme (China, 2016), Science and Technology Program of Guangdong (2018B030334001), Innovation Team Program of Guangdong Provincial Department of education (2018KCXTD006), and National Natural Science Foundation of Guangdong Province (2020ZDZX1059).

## Conflict of Interest

The authors declare that the research was conducted in the absence of any commercial or financial relationships that could be construed as a potential conflict of interest.

## Publisher's Note

All claims expressed in this article are solely those of the authors and do not necessarily represent those of their affiliated organizations, or those of the publisher, the editors and the reviewers. Any product that may be evaluated in this article, or claim that may be made by its manufacturer, is not guaranteed or endorsed by the publisher.

## Abbreviations

APRC: antipsychotic-related constipation
EA: electroacupuncture
SEA: sham-electroacupuncture
SBMs: spontaneous bowel movements
OTUs: operational taxonomic units
ROC: receiver operating characteristic
AUC: assessing the area under curve
ITT: intent-to-treat
LefSe: linear discriminant analysis effect size
LDA: linear discriminant analysis
PD: phylogenetic diversity.
