# Supplementary material for: Microbial Profiles of Patients With Antipsychotic-Related Constipation Treated With Electroacupuncture
Source: Front Med (Lausanne). 2021 Oct 14;8:737713. doi: 10.3389/fmed.2021.737713 (PMC8551555; doi:10.3389/fmed.2021.737713)
Supplement: Supplementary file 1 [file Data_Sheet_1.PDF]

**Supplementary material 1.**
Rome III diagnostic criteria for functional onstipation
1.Subjects must meet two or more of the following criteria:<br>①straining during at least 25% of defecations;<br>②lumpy or hard stools in at least 25% of defecations;<br>③sensation of incomplete evacuation for at least 25% of defecations;<br>④sensation of anorectal obstruction or blockage for at least 25% of defecations;<br>⑤manual maneuvers to facilitate at least 25% of defecation (support of fingers or pelvic floor);<br>⑥fewer than three SBMs per week.
2.It is rare to have loose stools without laxative use.
3.There is insufficient evidence for a diagnosis of irritable bowel syndrome(IBS).
