# Supplementary material for: Microbial Profiles of Patients With Antipsychotic-Related Constipation Treated With Electroacupuncture
Source: Front Med (Lausanne). 2021 Oct 14;8:737713. doi: 10.3389/fmed.2021.737713 (PMC8551555; doi:10.3389/fmed.2021.737713)

Figure S1 Acupoints

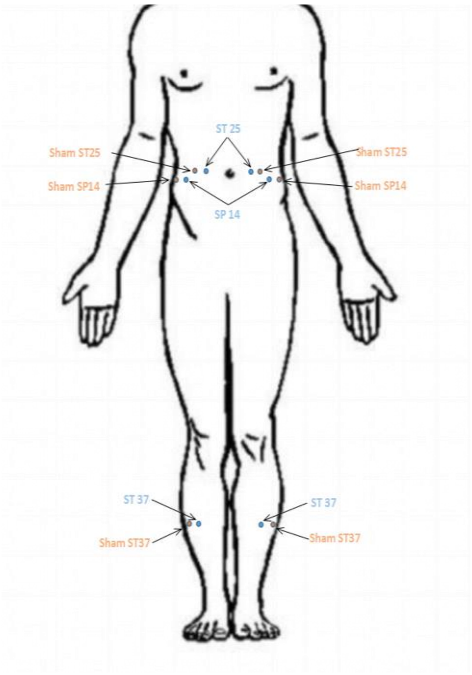

The location map of the acupoints used in this study in the human body. ST25 =Tianshu, SP14=Fujie, ST37=Shangjuxu, sham ST25=sham Tianshu, sham SP14=sham Fujie, sham ST37=sham Shangjuxu

Figure S2 Rarefaction Curves

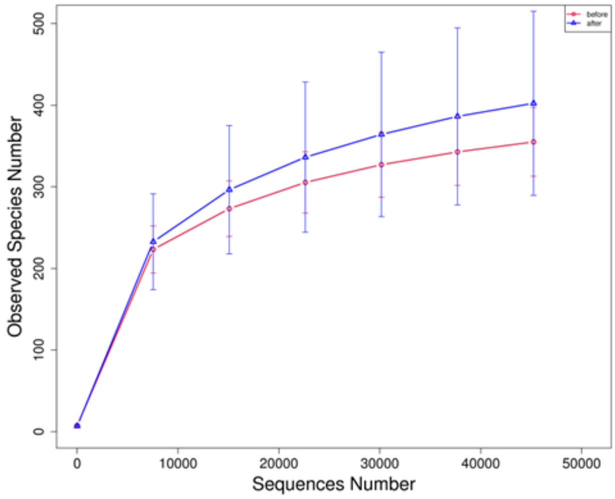

Figure S3 SBMs score in different age

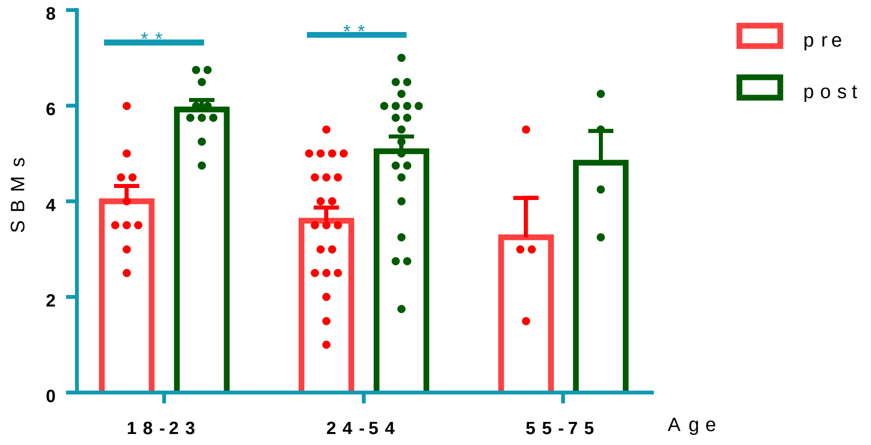

Supplement: Supplementary file 2 [file Data_Sheet_2.PDF]
